# Identification of cancer predisposition variants in apparently healthy individuals using a next-generation sequencing-based family genomics approach

**DOI:** 10.1186/s40246-015-0034-2

**Published:** 2015-06-20

**Authors:** Ioannis Karageorgos, Clint Mizzi, Efstathia Giannopoulou, Cristiana Pavlidis, Brock A. Peters, Zoi Zagoriti, Peter D. Stenson, Konstantinos Mitropoulos, Joseph Borg, Haralabos P. Kalofonos, Radoje Drmanac, Andrew Stubbs, Peter van der Spek, David N. Cooper, Theodora Katsila, George P. Patrinos

**Affiliations:** Department of Pharmacy, University of Patras, School of Health Sciences, University Campus, Rion GR-26504, Patras, Greece; Department of Physiology and Biochemistry, Faculty of Health Sciences, University of Malta, Msida, Malta; Clinical Oncology Laboratory, Division of Oncology, Department of Medicine, University of Patras, Patras, Greece; Complete Genomics Inc., Mountain View, CA USA; BGI-Shenzhen, Shenzhen, 51803 China; Institute of Medical Genetics, School of Medicine, Cardiff University, Cardiff, UK; The Golden Helix Foundation, London, UK; Department of Applied Biomedical Science, Faculty of Health Sciences, University of Malta, Msida, Malta; Department of Cell Biology and Genetics, School of Medicine and Health Sciences, Erasmus University Medical Center, Rotterdam, The Netherlands; Department of Bioinformatics, School of Medicine and Health Sciences, Erasmus University Medical Center, Rotterdam, The Netherlands

**Keywords:** Cancer predisposition, Family genomics, Next-generation sequencing, Genomic variants

## Abstract

Cancer, like many common disorders, has a complex etiology, often with a strong genetic component and with multiple environmental factors contributing to susceptibility. A considerable number of genomic variants have been previously reported to be causative of, or associated with, an increased risk for various types of cancer. Here, we adopted a next-generation sequencing approach in 11 members of two families of Greek descent to identify all genomic variants with the potential to predispose family members to cancer. Cross-comparison with data from the Human Gene Mutation Database identified a total of 571 variants, from which 47 % were disease-associated polymorphisms, 26 % disease-associated polymorphisms with additional supporting functional evidence, 19 % functional polymorphisms with in vitro/laboratory or in vivo supporting evidence but no known disease association, 4 % putative disease-causing mutations but with some residual doubt as to their pathological significance, and 3 % disease-causing mutations. Subsequent analysis, focused on the latter variant class most likely to be involved in cancer predisposition, revealed two variants of prime interest, namely *MSH2* c.2732T>A (p.L911R) and *BRCA1* c.2955delC, the first of which is novel. *KMT2D* c.13895delC and c.1940C>A variants are additionally reported as incidental findings. The next-generation sequencing-based family genomics approach described herein has the potential to be applied to other types of complex genetic disorder in order to identify variants of potential pathological significance.

**Electronic supplementary material:**

The online version of this article (doi:10.1186/s40246-015-0034-2) contains supplementary material, which is available to authorized users.

## Introduction

Cancer results from a multi-step cascade of somatic events involving the accumulation of both genetic and epigenetic changes at various genomic loci, under the influence of a variety of different environmental factors [1–5]. Single point mutations, small insertions/deletions, translocations, gene fusions, copy number changes, and loss of heterozygosity represent some of the somatic alterations frequently encountered in cancer [6] and which can lead to the increased expression of oncogenes or to the silencing of tumor suppressor genes. Genome-wide association studies (GWASs) have also identified genomic regions that appear to be associated with increased cancer risk [7–9]. It is to be expected that an improved knowledge of the genomic variants that predispose to tumor initiation, development, and progression will be advantageous in the context of informing treatment regimens. Numerous studies have been performed in an attempt to shed light on the complexity (and inter-individual variability) of the cancer genome and to examine the relationship between the possession of specific genomic variants and tumorigenesis [10, 11], often with ambiguous results.

The advent of next-generation sequencing (NGS) has provided unprecedented opportunities to decipher the cancer genome and to dissect the molecular etiology of cancer predisposition. This has been the primary goal of the International Cancer Genome Consortium (ICGC), following the initiatives of the Human Genome Project and the HapMap Consortium [12]. The comprehensive listings of genomic abnormalities (somatic mutations, abnormal gene expression, epigenetic effects) detected in tumors from 50 cancer types and/or subtypes of clinical and societal importance are being made available to the entire research community with minimal restrictions (http://www.icgc.org/icgc). In this context, the Catalogue of Somatic Mutations in Cancer (COSMIC; http://cancer.sanger.ac.uk/cancergenome/projects/cosmic) stores and displays our current knowledge of somatic mutations detected in human cancers, including information on publications and most notably, tissue samples (i.e., benign neoplasms, in situ and invasive tumors, recurrences, metastases) and cancer cell lines. In 2004, the Cancer Gene Census (http://cancer.sanger.ac.uk/cancergenome/projects/census) indicated that mutations in more than 1 % of genes may contribute to human cancer [13]. A total of 547 entries are currently reported, of which 90 % refer to somatic mutations in cancer, 20 % correspond to germline mutations that predispose to cancer, whereas 10 % refer to both somatic and germline mutations.

Genetic susceptibility to cancer is conferred both by inherited (germline) and tumor-specific (somatic) variants and as such, it is evident in most individuals, not just in those individuals with a personal or family history of cancer. Although the deleterious alleles of cancer risk genes are generally not highly penetrant, the presence of genetic susceptibility variants at multiple loci is generally assumed to increase an individual’s overall risk of cancer. It should be noted that, even in the case of highly penetrant cancer predisposition genes, such as *BRCA1* and *BRCA2*, population-based studies have revealed that about half of all heterozygous mutation carriers with incident cancers lack a family history of breast or ovarian cancer [14]. Further, individuals with no obvious indication of cancer risk from family history may nevertheless be at an increased risk of developing cancer [15]. NGS technologies have potentiated the analysis of whole genomes as a means to obtain a full picture of individual variomes [16]. Once whole genome and/or whole exome sequencing begins to take hold in routine clinical medicine, developing an understanding of the role of the detected sequence variations in guiding diagnostics and arriving at prognoses will be of paramount importance to the clinician, especially in the case of multifactorial disorders and cancer, where early detection of novel causative variants can be crucial for early disease diagnosis and health management. In the context of cancer, data interpretation will require an understanding of the heritable variation present in cancer risk-associated genes in healthy individuals. Currently, this knowledge is largely lacking.

Here, we propose a multi-step next-generation sequencing-based family genomics approach, piloted in 11 members of two families of Greek descent with no history of cancer, to identify genomic variants, particularly novel variants, that might predispose to various types of cancer. Such information could help in the assessment of personalized cancer-susceptibility risk from genome sequence data [17].

## Materials and methods

### Case selection, DNA isolation, and whole genome sequencing

**Fig. 1:**
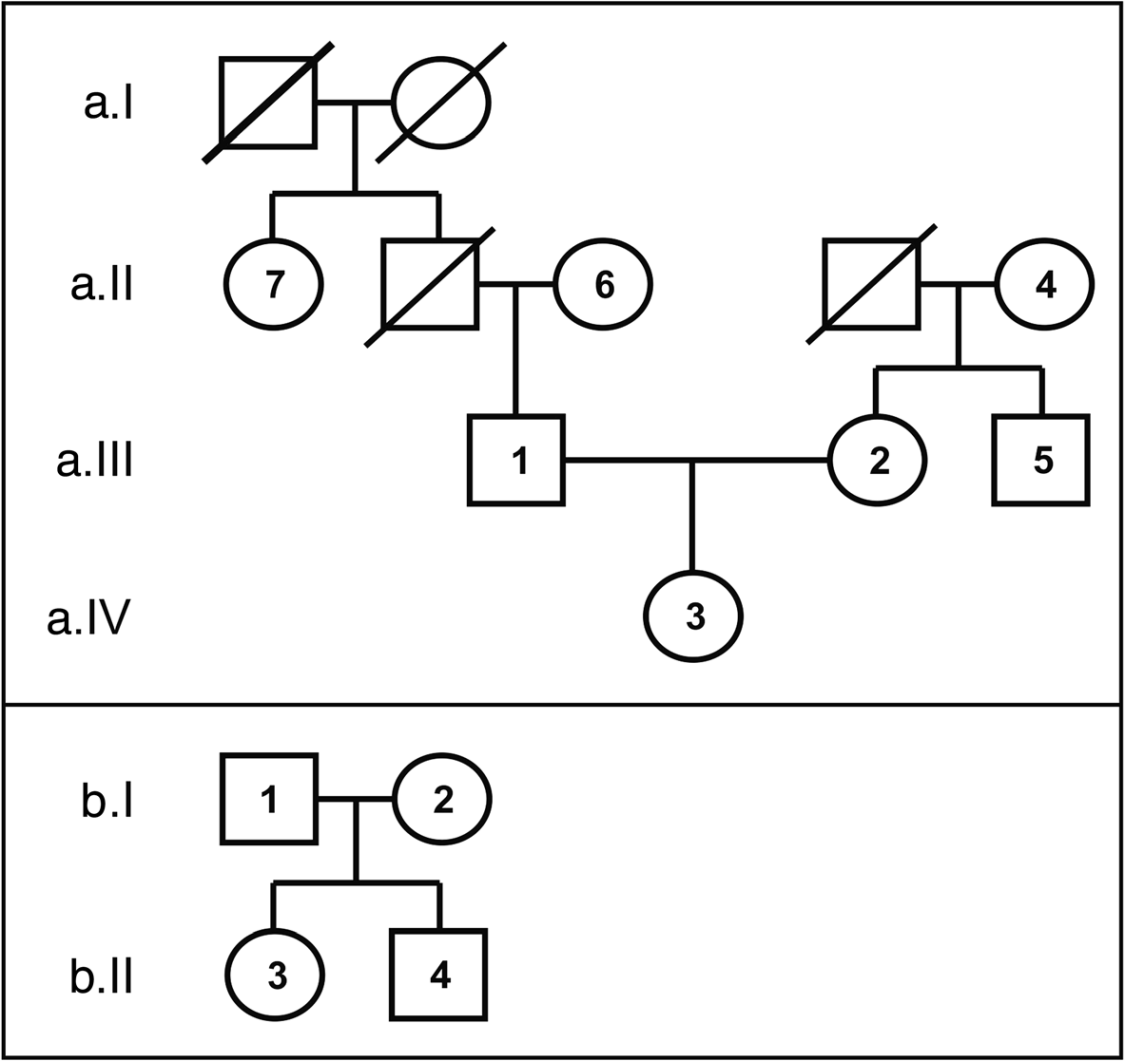
Pedigrees of the two families (*a*, *b*) of Greek descent whose genomes were analyzed

Eleven members of two unrelated families of Greek descent were recruited for this study (Fig. 1). All individuals consented to participate. A family-based design was employed rather than a population-based design, as the former is generally considered to be robust against population admixture and stratification and may yield both within- and between-family information [18]. None of the individuals tested had a family history of cancer (germline risk variants would not be anticipated as they are quite infrequent, although they cannot altogether be excluded). We aimed to identify cancer-susceptibility variants of low penetrance in these apparently healthy individuals, information that could help in the assessment of personalized cancer-susceptibility risk from genome sequence data. It was hoped that the application of the NGS approach might lead to the identification of novel cancer-susceptibility variants. Informed consent was obtained from all individuals who took part in this study.

Genomic DNA isolation was performed from saliva samples using the Oragene collection kit (DNA Genotek, Canada). Whole genome sequencing was performed using the proprietary DNA nanoball resequencing technology of Complete Genomics [19]. DNA sequencing coverage was 110×.

### Bioinformatics and in silico analyses

All variants were filtered according to the analysis required, using custom scripts and Complete Genomics Analysis Tools [CGA™ Tools]. Only high-quality call variants were included in the analysis (>93 %). Genomes were aligned with the hg19 reference genome and subsequently compared against the Human Gene Mutation Database, Professional version (HGMD v.2014.4; http://www.hgmd.org) records with respect to previously known variants in cancer predisposition genes. Subsequently, a non-redundant list of variants was generated from the 11 human genome sequences and subjected to text mining, using the following keywords: “Cancer,” “Adenoma,” “Carcinoma,” “Adenocarcinoma,” “Melanoma,” “Lymphoma,” “Leukemia,” “Glioma,” and “Glioblastoma.” Novel variants in genes that had previously been related to cancer predisposition were annotated with Annovar in Galaxy [20] and compared with NCBI dbSNP build 137 (http://www.ncbi.nlm.nih.gov/projects/SNP/snp_summary.cgi), 69 reference genomes from Complete Genomes (http://www.completegenomics.com/publicdata/69Genomes/), COSMIC v68 (http://cancer.sanger.ac.uk/cosmic/version68), and GWAS studies (http://www.genome.gov/gwastudies) to determine their novelty or otherwise.

To obtain a list of variants of potential functional significance, we employed protein variation effect analyzer (PROVEAN) v1.1.3 (PROVEAN human genome variants tool) that provides both scale-invariant feature transform (SIFT) [21] and PROVEAN [22] predictions for a given list of human genome variants as well as accessory information (dbSNP rs IDs, gene description, PFAM domain, GO terms, etc.). PROVEAN is able to make predictions for any type of protein sequence alteration, including single or multiple amino acid substitutions, deletions, and insertions [23]. In addition, we used CRAVAT (Cancer-Related Analysis of Variants Toolkit), a tool more specifically tailored to a cancer variant application, to facilitate the high-throughput assessment and prioritization of genes important for cancer tumorigenesis [24].

### Downstream molecular analysis

Selected novel variants were subsequently analyzed using a polymerase chain reaction (PCR)-based conventional Sanger resequencing approach and validated in a pool of ethnically matched 60 control samples to determine whether or not they constituted frequent variants.

## Results

### Next-generation sequencing-based family genomics

**Fig. 2:**
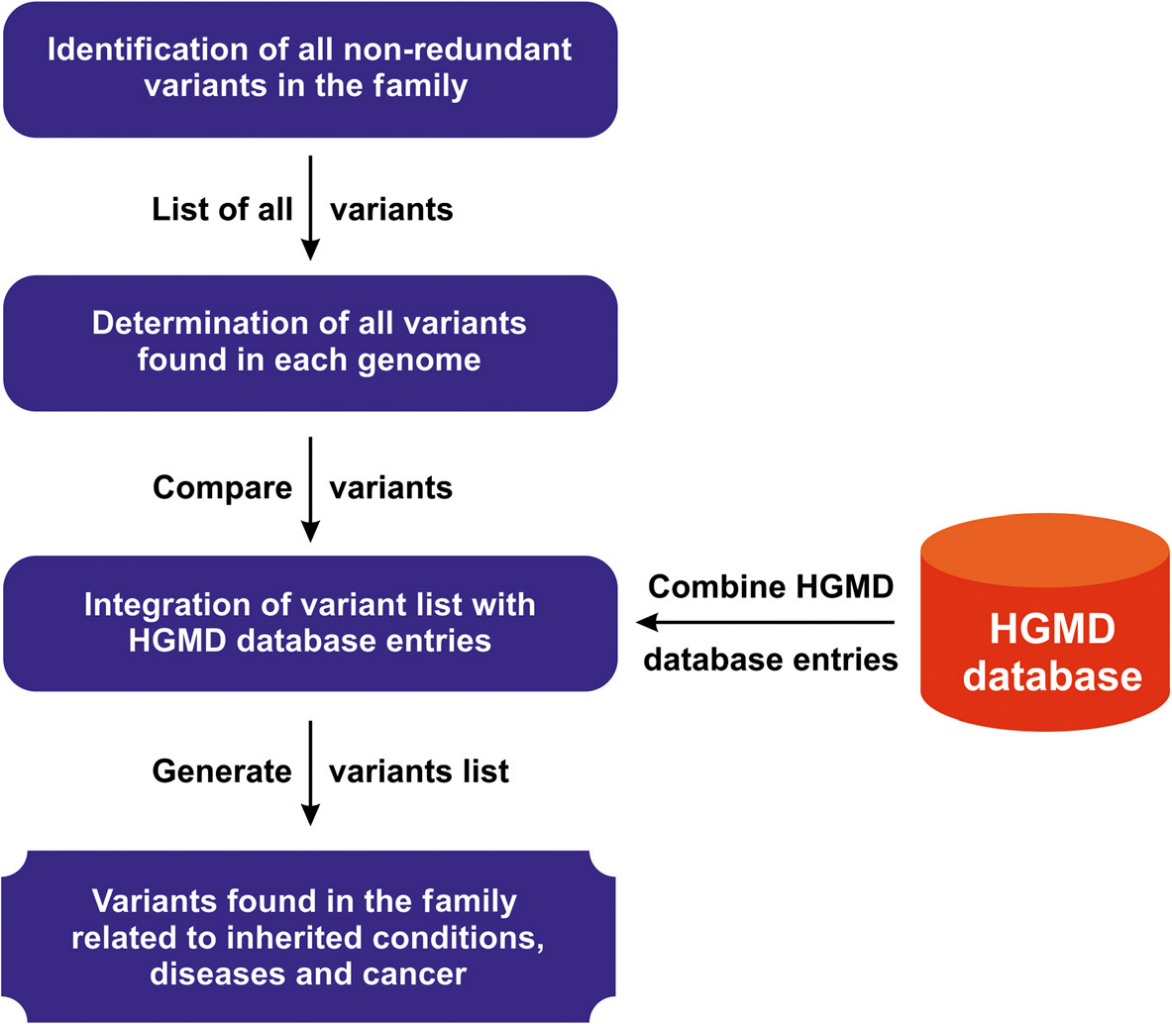
Outline of the concept of the family genomics approach implemented in this study

The family genomics approach adopted herein is depicted in Fig. 2. Eleven members from two unrelated Greek families had their genomes sequenced. None of the individuals from the families selected had any history of cancer, as indicated by the participants. Our family-based approach was adopted in order to identify known cancer risk variants as well as novel variants of low penetrance—although highly penetrant genes cannot be excluded—in healthy individuals, information that could help in the assessment of personalized cancer-susceptibility risk from genome sequence data.

A list of 571 non-redundant genomic variants that might predispose family members to cancer was identified after cross-comparison with HGMD entries (Additional file 1: Table S1) and further categorized by genome/family member on the basis of the text mining terms used. To further address individual cancer risk, clinical impact data were also incorporated accompanied by GWAS data, allele frequencies (Complete Genomics 69 diversity file), 1000 Genomes Project data as well as the input of COSMIC v68.

### Analysis of 11 genomes reveals a large number of potential cancer predisposition variants

**Fig. 3:**
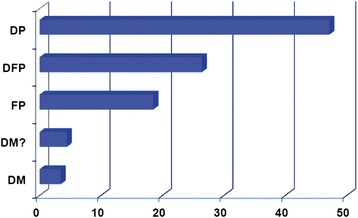
Summary of the genomic variants identified, grouped per variant classes in the HGMD database. HGMD variant classes were as follows: *DP* disease-associated polymorphisms, *DFP* disease-associated polymorphisms with additional supporting functional evidence, *FP* functional polymorphisms with in vitro/laboratory or in vivo supporting evidence but no known disease association *DM* disease-causing mutations, *DM?* putative disease-causing mutations but where there is some residual doubt as to pathological significance

**Fig. 4:**
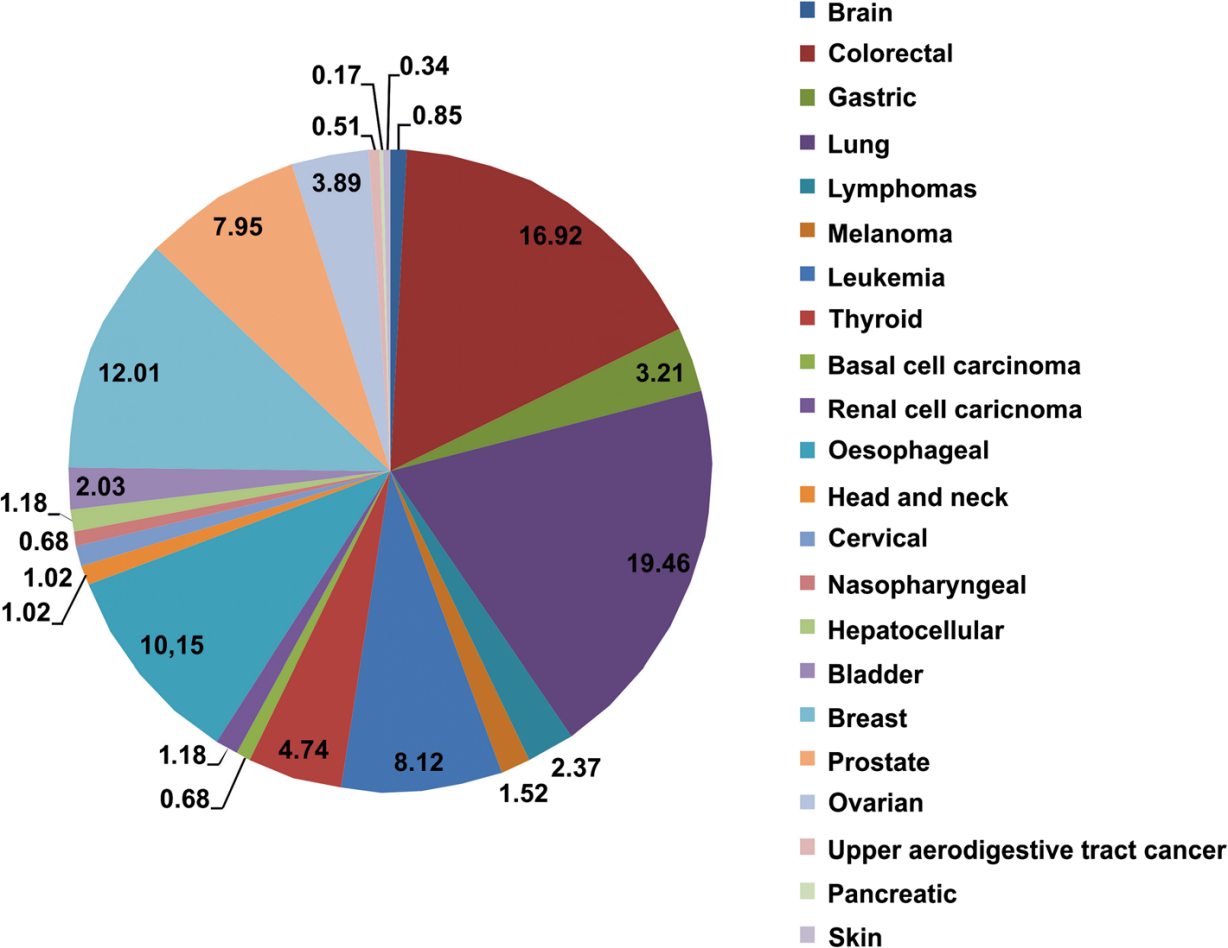
Variants identified by cancer type in all 11 genomes from the two families under study

Cross-comparison with data from the Human Gene Mutation Database identified a total of 571 heritable variants that had previously been reported to be associated with cancer predisposition (Additional file 1: Table S1). Taking into account the variant classes included in the HGMD dataset, the distribution of our 571 variants was 47 % DP (disease-associated polymorphisms), 26 % DFP (disease-associated polymorphisms with additional supporting functional evidence), 19 % FP (functional polymorphisms with in vitro/laboratory or in vivo supporting evidence but no known disease association), 4 % DM? (putative disease-causing mutations but where there is some residual doubt as to pathological significance), and 3 % DM (disease-causing mutations) (Fig. 3). Subsequent analysis focused on the DMs as the variant class most likely to be involved in cancer predisposition (Table 1). Cancer predisposition to various cancer types was revealed including brain, head and neck, bladder, breast, lung, gastric, prostate, colorectal, ovarian, thyroid, oral, hereditary non-polyposis colorectal cancer (HNPCC), esophageal, upper aerodigestive tract, pancreatic, and skin cancer. The variants identified were also distributed over a broad range of tumor (tissue) types: gliomas/glioblastomas, adenomas (colorectal), lymphomas (non-Hodgkin’s), adenocarcinomas (lung, gastric), melanomas, leukemias, and carcinomas (thyroid, basal cell, renal cell, esophageal, cervical, nasopharyngeal, hepatocellular). The majority of the cancer predisposition variants related to lung, colorectal, and breast cancer (Fig. 4).

**Table 1:** The DM (disease-causing mutations) and DM? (putative disease-causing mutations but where there is some residual doubt as to pathological significance) mutations identified in each family

	Cancer type	HGMD tag
Family A, annotated variants		
dbsnp.129:rs56250729	Prostate cancer	DM?
dbsnp.125:rs28756990	Endometrial cancer	DM?
dbsnp.131:rs77067228	Cancer	DM?
dbsnp.89:rs1799966	Breast and/or ovarian cancer	DM/DM?
dbsnp.113:rs4986852	Breast cancer	DM?
COSMIC:mut:148278;dbsnp.86:rs799917	Breast and/or ovarian cancer	DM
dbsnp.89:rs1799950	Breast cancer	DM
dbsnp.98:rs2229995	Adenomatous polyposis coli	DM?
dbsnp.127:rs41545019	Colorectal cancer	DM?
dbsnp.98:rs1805324	Colorectal cancer, non-polyposis	DM?
dbsnp.129:rs61753720	Acute lymphoblastic leukemia	DM
Family B, Annotated variants		
dbsnp.126:rs33927012	Medullary thyroid carcinoma	DM?
dbsnp.98:rs2229992	Adenomatous polyposis coli	DM
dbsnp.76:rs41115	Adenomatous polyposis coli	DM
dbsnp.79:rs169547	Breast cancer	DM?
dbsnp.103:rs3092994	Breast cancer	DM?
dbsnp.89:rs1799966	Breast cancer	DM
dbsnp.86:rs1060915	Breast cancer	DM
dbsnp.125:rs28897689	Breast and/or ovarian cancer	DM
COSMIC:mut:148278;dbsnp.86:rs799917	Breast and/or ovarian cancer	DM
dbsnp.60:rs16940	Breast cancer	DM

Four variants that have not so far been annotated in either dbSNP or the 1000 Genomes Project/exome variant server data were considered to be of prime interest; *MSH2* c.2732T>A (p.Leu911Arg), *BRCA1* c.2955delC, and *KMT2D* c.13895delC and c.1940C>A variants (Additional file 1: Table S1). The *MSH2* c.2732T>A (p. Leu911Arg) variant is novel as it is not present in HGMD. The *KMT2D* c.13895delC and c.1940C>A variants are reported herein as incidental findings. All the putative cancer-associated mutations were also considered in terms of assessing personalized cancer-susceptibility risk from genome sequence data.

**Fig. 5:**
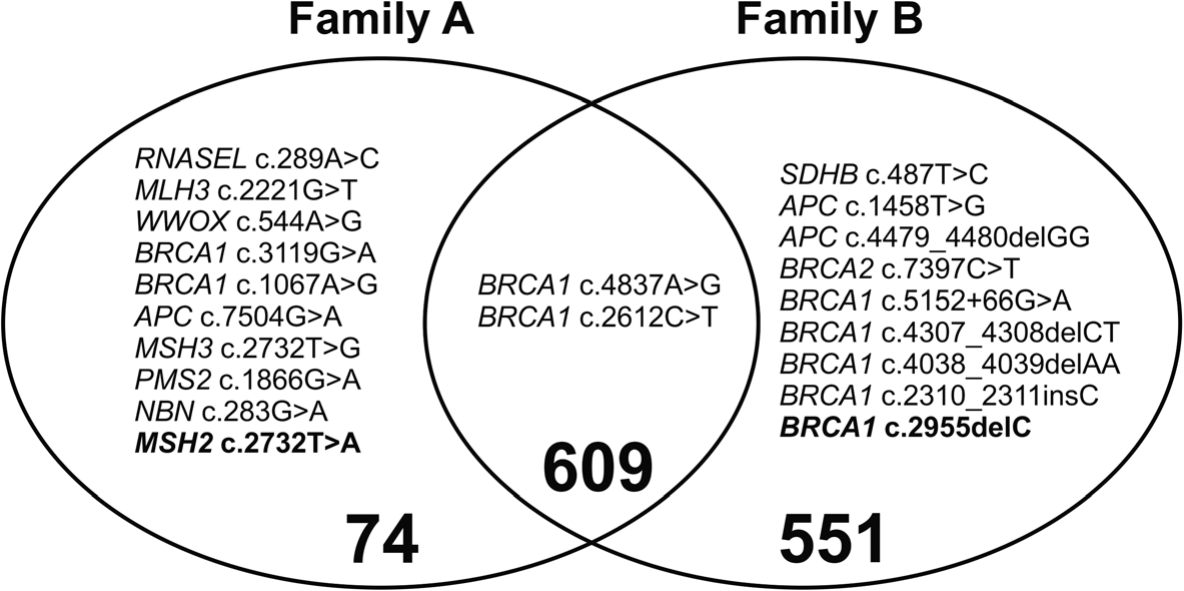
Variants identified per family in all 11 genomes from the two families under study. The DM (disease-causing mutations) and DM? (putative disease-causing mutations but where there is some residual doubt as to pathological significance) mutations identified as well as our two variants of prime interest, *MSH2* c.2732T>A and *BRCA1* c.2955delC, are shown

It became evident that, among the 571 cancer risk-associated variants identified, some were common between the two families considered, whereas others were unique (Fig. 5). In particular, 609 variants were found in both families, while 74 variants were unique to family A and 551 variants were only found in family B. Commenting on the unique variants obtained, family B comes from northern Greece, a quite distant location from Athens (300.13 km) where family A is from, implying a different genetic origin.

### In silico and replication analyses

To ascertain whether the variants of interest (*MSH2* c.2732T>A (p. L911R) and *BRCA1* c.2955delC) have functional significance, in silico analysis was performed using the SIFT and PROVEAN algorithms [21, 22]. As depicted in Table 2, SIFT analysis yielded a p.L911R substitution (*MSH2* c.2732T>A) which was predicted to be “deleterious” to protein function with a score equal to or less than 0.05, implying that this could be a pathologically relevant variant. Moreover, PROVEAN’s scoring scheme has been shown to perform well in separating disease-associated variants from common polymorphisms [25]. It is thought that non-synonymous SNPs exhibiting a deleterious effect on function may have become established, being potentially beneficial under some historical conditions, although today they may only be found at low frequency [26]. Using a polymerase chain reaction (PCR)-based conventional Sanger resequencing approach, *MSH2* c.2732T>A (p. L911R) was subsequently investigated in a pool of ethnically matched control samples and determined to be an infrequent variant in agreement with PROVEAN’s scoring scheme.

**Table 2:** SIFT PROVEAN analysis outcome of the two variants of prime interest identified in the 11 family members

HUGO gene symbol	Chromosome	HGVS description of variant	HGMD tag	Aminoacid position	Reference residue	Alternative residue	Variation type	PROVEAN prediction		SIFT prediction	
								Score	Prediction	Score	Prediction
*MSH2*	2	c.2732T>A	NA	911	L	Q	Single AA Change	−4.14	Deleterious	0.001	Damaging
*BRCA1*	17	c.2955delC	DM	986			Frameshift	NA	NA	NA	NA

Source: PROVEAN v1.1.3 (PROVEAN human genome variants tool, http://provean.jcvi.org/genome_submit_2.php?species=human).PROVEAN was developed in order to predict whether a given protein sequence variation (single or multiple amino acid substitutions, micro-insertions, micro-deletions) affects protein function. To achieve this, PROVEAN introduces a delta alignment score based on the reference and variant versions of a protein query sequence with respect to sequence homologs (NCBI NR protein database through BLAST, http://www.ncbi.nlm.nih.gov/). The default score threshold was set at −2.5 for binary classification (deleterious<−2.5 vs. neutral>−2.5). Similarly (through PSI-BLAST, http://blast.ncbi.nlm.nih.gov/Blast.cgi?CMD=Web&PAGE=Proteins&PROGRAM=blastp&RUN_PSIBLAST=on), SIFT (http://sift.jcvi.org/) may be applied to naturally occurring non-synonymous polymorphisms. SIFT score ranges from 0 to 1. A SIFT score of ≤0.05 corresponds to a “damaging” prediction, whereas a SIFT score >0.05 predicts that the variant is likely to be “tolerated”

*NA* not available (where the precomputed score was not available in the database, the precomputed homologous protein identifiers for the query protein were retrieved in order to bypass the BLAST search and clustering, and the score was computed based on the homologs)

CRAVAT analysis (Table 3) was performed in two ways: firstly by querying a functional effect and then by proceeding with cancer driver analyses: a general analysis and subsequently a targeted one, choosing “colon” as the tissue type of interest, on the basis of the findings depicted in Table 1 regarding *MSH2*, as well as literature-derived evidence supporting its association with microsatellite instability and hereditary non-polyposis colorectal cancer. According to the functional analysis outcome, *MSH2* c.2732T>A (p.L911R) and *BRCA1* c.2955delC were observed in various cancer types in COSMIC (grouped by primary site). Cancer driver gene hits (tumor suppressor genes), according to Vogelstein and coworkers [27], were also obtained for *BRCA1* c.2955delC and *MSH2* c.2732T>A (p.L911R). Notably, the frameshift variant of *BRCA1* was accompanied by a TARGET (*T*umor *A*lterations *R*elevant for *GE*nomics-driven *T*herapy) drug association hit (http://www.broadinstitute.org/cancer/cga/target). TARGET genes, when somatically altered in cancer, are linked to a clinical action (prediction of therapy response/ resistance, prognosis, diagnosis).

**Table 3:** CRAVAT functional analysis outcome of the two variants of prime interest identified in the 11 family members

HUGO gene symbol	HGVS description of variant	HGMD tag	Sequence ontology	Driver genes	Target	Occurrences in COSMIC by primary sites (gene mutated)
*MSH2*	c.2732T>A	NA	Missense variant	TSG	NA	Cervix(1), large intestine (106), autonomic ganglia (2), central nervous system (2), liver(1), small intestine (1), haematopoietic and lymphoid tissue (7), endometrium (18), urinary tract (1), lung (14), breast (7), skin (4), stomach (1), esophagus (1), ovary (4), NS (2), prostate (2), kidney (6), pancreas (1)
*BRCA1*	c.2955delC	DM	Frameshift deletion	TSG	PARP inhibitor	Cervix (1), large intestine (66), stomach (8), central nervous system (2), pancreas (1), meninges (1), haematopoietic and lymphoid tissue (3), endometrium (23), urinary tract (5), lung (42), liver (5), skin (6), oesophagus (4), ovary (39), NS (2); prostate (2), kidney (5), breast (33)

Source: CRAVAT tool. In the case of *MSH2*, cancer driver analyses were also performed, selecting “colon” as the tissue type of interest. A driver score of 0.27 was obtained (a driver score close to zero means an increased probability of the mutation being a cancer driver)

*TSG* tumor suppressor gene, *NA* not available

## Discussion

The etiology of cancer is highly complex, being characterized by a strong genetic component (a large number of somatic as well as germline variants have been associated with different cancer types), while multiple environmental factors contribute to disease susceptibility. In 1974, Anderson stated that the two- to threefold excess observed in first-degree relatives of cancer patients is not indicative of strong genetic effects. They are more suggestive of the involvement of many genes with small effects acting in concert with environmental or nongenetic factors with larger and more important effects [28]. Although according to Peto (1980) this was a statistical fallacy [29], it seems that the overall statement is correct since current epidemiological evidence is supported by the discovery of low-penetrance genes that predispose to the majority of cancer types. Under the polygenic model, a large number of alleles each conferring a small genotypic risk (perhaps a relative risk of 1.5–2.0) combine additively or multiplicatively to confer a range of susceptibilities in the general population [30]. Hence, individuals are at elevated risk in the presence of the combined effects of several susceptibility alleles. This implies that unless a large number of the relevant alleles have been identified for a susceptible group, this group cannot be targeted for early screening or prophylactic therapy. Reduced (or incomplete) penetrance may be also of importance, in an attempt to understand why healthy individuals can harbor several potentially disadvantageous variants in their genomes without suffering any obvious ill effects. Next-generation sequencing of entire exomes or genomes of apparently normal healthy individuals from the general population supports the view that reduced penetrance is actually a widespread phenomenon in human genetics [31]. We believe that large-scale sequencing and genotyping studies of apparently healthy individuals could provide a powerful new approach to understanding the penetrance of pathological mutations/genotypes.

The advent of next-generation sequencing has provided unprecedented opportunities to decipher the cancer genome and to delineate the molecular etiology underlying cancer predisposition, similar to the numerous applications of next-generation sequencing in the elucidation of the molecular basis of rare diseases [23] and pharmacogenomics [32]. Herein, we pursued a next-generation sequencing-based family genomics approach in 11 members of two families of Greek descent in order to identify genomic variants that might predispose family members to cancer. None of the individuals tested had a family history of cancer (germline genomic variants would not be anticipated), and hence, we aimed to identify genomic variants, particularly novel variants, that might predispose currently asymptomatic individuals to various types of cancer.

A total of 571 variants previously shown to be associated with cancer predisposition were identified. We assessed both non-synonymous and synonymous variations, since the latter can impact the mRNA phenotype (whether via transcription, splicing, mRNA transport or translation) thereby rendering the synonymous mutation non-silent [33]. In a recent study that focused on germline mutations in cancer-susceptibility genes, the authors identified non-synonymous genomic variations in 158 genes causally implicated in carcinogenesis, using high-quality whole genome sequences from an ancestrally diverse cohort of 681 healthy individuals, none of whom were first-degree relatives [34]. As in our study, all individuals were found to carry multiple variants with the potential to impact cancer-susceptibility. Bodian and coworkers, however, focused on the detailed analysis of a selected subset of five clinically important cancer genes (*BRCA1*, *BRCA2*, *KRAS*, *TP53*, and *PTEN*), highlighting differences between germline variants and reported somatic mutations, reporting on their allele frequencies by ancestry. Differences between ancestry groups (African, African-European, Central Asian, East Asian, European, Hispanic, others) were also reflected in the number of cancer-gene variants as well as the number of deleterious variants per individual. In the case of *TP53* c.215C>G (p.P72R), *BRCA1* c.2612C>T (p.P871L), *ERBB2* c.3508C>G (p. P1170A), and *FLT3* c.680C>T (p.T227M), the minor allele in one population was shown to be the major allele in another.

Our study revealed four variants—namely, *BRCA1* c.2955delC, *MSH2* c.2732T>A (p.L911R), and *KMT2D* c.13895delC and c.1940C>A—that have not been annotated in either dbSNP or the 1000 Genomes Project/exome variant server data. It should be noted that there is no HGMD entry for *MSH2* c.2732T>A (p.L911R), and hence, it may be considered to be novel. Inherited mutations in *BRCA1* are well known to confer an increased lifetime risk of developing breast or ovarian cancer. *BRCA1* is a tumor suppressor gene that is involved in the maintenance of genome stability (homologous recombination pathway for double-strand DNA repair) and hence is of paramount importance in hereditary breast and ovarian cancers. However, the identification of an evidently detrimental *BRCA1* variant in a healthy individual is not unlikely [35]. This may raise awareness regarding the use of next-generation sequencing in oncology.

*MSH2* c.2732T>A (p.L911R) was predicted by PROVEAN to be “deleterious” to protein function, implying that this is a case of a disease-associated variant (amino acid variant that deviates from the frequently occurring residue) and was assigned a driver mutation score close to zero (a driver score close to zero implies increased likelihood of the mutation being a cancer driver) for colon cancer (by CRAVAT). Using a PCR-based conventional Sanger resequencing approach, this variant was verified as a non-frequent one in agreement with PROVEAN’s scoring scheme, separating disease-associated variants from common polymorphisms [25]. The DNA mismatch repair protein Msh2 (also known as MutS protein homolog 2, MSH2) is encoded by the *MSH2* gene (tumor suppressor gene), which is located on chromosome 2. Mutations in the *MSH2* gene are associated with microsatellite instability and cancer (hereditary non-polyposis colorectal cancer (HNPCC)). MSH2, as a heterodimer with MSH6, forms the human MutSα mismatch repair complex. MSH3 is also a dimerization partner of MSH2 towards the formation of the MutSβ DNA repair complex. MSH2 participates in several DNA repair processes, such as transcription-coupled repair [36], homologous recombination [37] as well as base excision repair [38]. The amino acid change consequent to the identified missense variant is located within the protein domain that serves to allow MSH2 to interact with MSH6/MSH3 [38]. Although the crystal structure of an MSH2/MSH6 heterodimer in complex with a DNA fragment has been solved [39, 40], the region containing the p.L911 residue was not resolvable. For this reason, we were unable to visualize the effect that the p.L911R might have on the protein.

*KMT2D* c.13895delC is a mutation, which has previously been associated with Kabuki (Niikawa-Kuroki) syndrome (autosomal dominant) with a particularly severe congenital phenotype: left-sided cardiac abnormalities, facial dysmorphisms, skeletal, renal and anorectal malformations, and hypertricosis [41, 42]. Interestingly, this syndrome has also been associated with cancer predisposition in children. The KMT2D protein encoded by this gene is a histone methyltransferase that methylates the Lys-4 position of histone H3 [43]. This notwithstanding, both *KMT2D* variants obtained herein (c.13895delC and c.1940C>A) are reported as incidental findings. There is currently an ongoing debate about reporting incidental findings in the context of genomic testing. American College of Medical Genetics and Genomics (ACMG) recommendations have been established and approximately 1 % of genome sequencing reports are expected to include an incidental variant that falls within the ACMG recommendations [44]. Although the aforementioned *KMT2D* variants were not given in the ACMG list, we chose to report and note them as incidental findings that could require further evaluation.

Our study has a number of limitations. Firstly, functional analysis of the *BRCA1* c.2955delC and *MSH2* c.2732T>A (p.L911R) variants was not performed. Secondly, although the SIFT-based in silico analysis provides an assessment of the likely pathogenicity of these novel variants, it cannot fully replace functional assays. Furthermore, our study considered only those genomic variants previously associated with cancer predisposition and that had been logged in HGMD Professional at the time of our analysis. Since this resource is frequently updated, the bioinformatics analysis of these genomes should be replicated periodically to exploit any new variants reported therein. Looking to the future, whole genome sequencing should ideally be performed once, e.g., at birth, with data analysis being frequently replicated thereafter in order to exploit the wealth of genomic knowledge that is continually becoming available. Lastly, family history is of central importance in medical/clinical practice, since it reflects both genetic and environmental exposures within families. Herein, none of the individuals reported had a family history of cancer (germline genomic variants would not be anticipated), rendering it highly questionable as to whether they would meet the normal criteria for genetic counseling. Incidental findings and reduced (incomplete) penetrance in cancer complicate decision-making even further. Nowadays, genomic data are hardly integrated in medical decision-making in cancer, given its complexity and as educational initiatives and support from specialists are also lacking [45]. In this context, a community knowledge base has been proposed by Good and coworkers (2014) to facilitate collaborative contributions and open discussions on genomic events [46].

In relation to the diagnosis and prognosis of cancer patients, data interpretation requires an understanding of the variation in cancer risk-associated genes in healthy individuals. This knowledge is still largely lacking. Herein, we followed a family-based genomics approach in healthy individuals to assess cancer risk via the identification of genomic variants, particularly novel ones that might predispose to various types of cancer. A crude assessment of the potential extent of the genome-wide cancer-susceptibility burden in normal healthy individuals was also an objective of this study, taking into account all the (putative) risk-associated mutations obtained. As whole genome and/or whole exome sequencing approaches begin to be recruited into clinical care, our understanding of detected sequence variations on diagnosis (and prognosis) needs to become more readily accessible to the clinician. This is not a trivial undertaking, especially as the polygenic model proposes that an individual’s cancer risk is the net outcome of the presence of multiple variants and environmental factors [47]. The use of next-generation sequencing is expected to play a crucial role in delineating an individual’s variome as well as providing the means to identify novel variants to improve therapeutic modalities. Signature-based drug-repositioning methods are also known to make use of gene signatures to uncover unknown mechanisms of action of molecules and drugs by coupling the significantly changed genes to computational approaches [48]. As whole genome sequencing services become more accurate in delivering clinical-grade genome sequences and whole genome sequencing costs continue to decline, it is expected that this approach will gradually assume an integral role in genomic medicine. The next-generation sequencing-based family genomics approach employed here could be readily replicated for other types of disorders to identify causative variants and/or in the context of signature-based drug-repositioning methods.

## Additional file

Additional file 1: Table S1. Known and novel cancer predisposition variants identified in all 11 members of the two families. A list of 571 non-redundant genomic variants that might predispose family members to cancer was identified after cross-comparison with HGMD entries and categorized by genome/family member on the basis of the text mining terms used.
